# Magnetic core-shell nanoparticles for Hyperthermia: A numerical study of soft and hard core-shell magnetic materials in liver tissue based on dual phase lag model

**DOI:** 10.1016/j.bbrep.2025.102084

**Published:** 2025-06-27

**Authors:** M. Moshtagh, M. Servatkhah, S. Hosseini, Gh Solookinejad

**Affiliations:** a Department of Physics, Marvdasht Branch, Islamic Azad University, Marvdasht, Iran; b Department of Physics, Nanotechnology Research Center, Marvdasht Branch, Islamic Azad University, Marvdasht, Iran

**Keywords:** Magnetic nanoparticles, Hyperthermia, Core-shell, Dual-phase-lag, Liver tissue

## Abstract

In this article, local hyperthermia using core-shell magnetic nanoparticles based on soft and hard magnetic ferrite phases, comprising Zn _0.4_Co _0.6_Fe _2_O _4_ @Zn _0.4_ Mn _0.6_ Fe _2_O _4_, under the influence of an AC magnetic field, has been numerically investigated to simulate heat distribution and tumor destruction in liver tissue.

It is observed that the dual-phase-lag (DPL) model predicts the maximum temperature lower than both the Pennes bioheat and the single-phase-lag (SPL) model. In addition simulation of temperature distribution over time considering different core-shell nanoparticles in AC magnetic field, has been performed using DPL model. The highest temperature is related to Zn _0.4_ Co _0.6_ Fe _2_O_4_ @Zn _0.4_ Mn _0.6_ Fe _2_O_4_ and the lowest temperature is related to MnFe_2_O_4_.We have concluded that these combinations maximize the properties of magnetic nanoparticles and have higher SLP values and more power dissipation of magnetic nanoparticles compared to magnetic nanoparticles of MnFe_2_O _4_, MnFe_2_O_4_ @ CoFe_2_O_4_ and CoFe_2_O_4_ @MnFe_2_O4.

Two-dimensional temperature distribution simulation over time in liver tissue has been performed using DPL model to quantitatively investigate the tumor temperature in different locations. The results show that temperature curves is a Gaussian-like distribution. The temperature curve is symmetric around the y axis. Temperature is maximum at the center of the tumor and decreases radially outward.

## Highlights

• Investigation of local hyperthermia using core-shell magnetic nanoparticles for simulating heat distribution in liver tissue and tumor destruction.
• Introduction of the dual-phase-lag (DPL) model to capture micro tissue-scale responses in time and space, offering a more accurate representation of heat transfer in biological tissues.
• Comparison of simulation results using Fourier, single-phase-lag (SPL), and dual-phase-lag (DPL) heat transfer equations, highlighting the advantages of the DPL model in predicting temperature distribution.
• Evaluation of various core-shell magnetic nanoparticle combinations and their effects on temperature distribution and tumor destruction under the influence of an AC magnetic field.
• Demonstration of the potential of core-shell magnetic nanoparticles, particularly Zn0.4Co0.6Fe2O4@Zn0.4Mn0.6Fe2O4, in maximizing heat generation and improving cancer treatment outcomes through hyperthermia.
• These research highlights underscore the innovative nature of our study and its potential impact on advancing hyperthermia treatment for cancer.

## 1 Introduction

After heart disease, cancer stands as the primary cause of death. This disease arises from the uncontrolled growth of cells, and if it progresses and spreads within a patient's body, it can ultimately lead to their demise. Among the cancer treatment methods (surgery, radiotherapy, and chemotherapy), hyperthermia plays a crucial role. Hyperthermia involves raising the body temperature intentionally to destroy cancer cells [1]. Recent advancements aim to selectively apply heat to cancer cells without harming healthy ones—a technique known as local hyperthermia [2,3] Various modalities contribute to this heat generation: microwaves, lasers, ultrasound, and magnetic nanoparticles [4]. In fact, magnetic heat therapy (MHT) has emerged as an alternative to traditional chemotherapy and radiation [5]. Minimally invasive thermal therapies, including laser treatments and the use of magnetic nanoparticles, are now emerging as local treatment options for a range of medical conditions. These conditions span from cancer and cardiovascular diseases to neurological disorders and chronic pain, all of which involve exposure to high-temperature heat sources within biological tissue [[6], [7], [8]]. One of the primary goals in nanotechnology is targeted drug delivery, which involves attaching drugs to nanoparticles for precise delivery to specific cells, minimizing harm to surrounding tissues. Utilizing magnetic nanoparticles (MNPs) and an external magnetic field enables intelligent drug delivery to targeted tissues. Superparamagnetic and ferromagnetic nanoparticles are key types of MNPs that enhance a magnetic field when exposed to one but lose their magnetic properties when the field is removed. Recent research focuses on enhancing the magnetic properties, particularly magnetic energy, of these systems by increasing the proportion of transition metals in permanent magnets during nanocomposite fabrication and employing exchange coupling of hard and soft magnetic phases [[9], [10], [11]].

The most suitable nanoparticles for this type of treatment are different ferromagnetic-containing elements like Fe, Ni, Co, Mg, Zn, etc. [5]. The most common materials used in hyperthermia are magnetic nanoparticles of iron oxides, such as magnetite (Fe_3_O_4_) and hematite (Fe_2_O_3_) [1,4].

Many nanoparticles with a core@shell structure have more applications. The core has a different surface area, structure, or composition, and therefore it can be adjusted with different properties. These systems are of great interest for both production and application purposes [[12], [13], [14]]. In particular, by fine-tuning the magnetic properties within the core-shell structure, SLP can be optimized for a specific particle size. These nanoparticles find applications in various biomedical fields, including hyperthermia-based cancer treatment [15]. In the case of superparamagnetic nanoparticles, it has been observed that the optimal size (approximately 22 nm) corresponds to the maximum SLP [[15], [16], [17]]. As previously mentioned, thermotherapy is considered as a type of cancer treatment. [8,18]. Accurately estimating temperature distribution is crucial to minimize damage to the surrounding healthy cells [8,19]. Tzou introduced the advanced dual-phase model. This dual-phase delay model can encompass all the proposed models by adjusting two key variables: the heat flux time delay (Ƭq) and the temperature gradient time delay (Ƭt) [20].

Researchers used Laplace transform analysis with experimental temperature data and time sequences to determine thermal damage and temperature in laser-irradiated living tissue. They also investigated how blood circulation and thermal relaxation time affect skin tissue temperature and the extent of thermal injury [21].

The Pence equation, rooted in Fourier's law, Is Derived from heat transfer principles, However, its assumption of infinitely rapid heat transfer has faced substantial criticism [6,22]. Heat spreads at a limited rate within biological tissues due to thermal delays and non-Fourier behavior, particularly in tissues with inhomogeneous internal structures [6,[23], [24], [25]]. This issue was raised by proposing a heat conduction model with a single-phase delay (SPL). [26,27]. In Jaunich's proposed SPL model, the distribution of temperature in tissue when exposed to laser radiation was investigated. The conclusion drawn from this study is that the SPL model provides greater accuracy than the PBH model [8,28].

Researchers used the finite element method to solve the biological heat transfer equation with relaxation time in cylindrical living tissue and validated their numerical solution against experimental data. This comparison indicated that their mathematical model is effective for assessing bioavailability transfer in living tissue. Additionally, studies have explored the numerical estimation of a nonlinear parabolic bioheat equation under various boundary conditions for tumor cell treatment. [30, 29]

After the development of the single-phase-lag (SPL) model, researchers introduced the non-Fourier dual-phase-lag (DPL) model by incorporating a second-phase delay. In the DPL model, these phase delays are denoted as Ƭ _q_ and Ƭ _t_. Specifically, Ƭ _q_ represents the time delay associated with heat flux propagation, while Ƭ_t_ corresponds to the time delay governing temperature changes. The DPL model enables capturing microtissue responses at both temporal and spatial scales. [8,20,28,31,32]. Multiple studies have been conducted in the field of dual phase delay (DPL):

Researchers have performed analytical solutions of the dual-phase bioheat equation with various boundary conditions for treating cancer cells. Researchers have also proposed a method for determining a numerical solution for thermal injury in living tissues using a delayed nonlinear two-phase model. The numerical results obtained from this technique were compared with existing experimental studies to confirm the accuracy of the numerical computations [33,34].In another study, mathematical models of biological heat transfer have been solved based on a nonlinear two-phase model using the finite element method to investigate transient phenomena in spherical tissue due to the effect of a laser heat source [35]. Zhu et al. established light energy deposits in the tissue and, using the diffusion theorem, created models of speed processes for thermal damage caused by them. The equation of bioheat conduction for the dual-phase delay of DPL was solved analytically, and its results were compared with experimental data, proving the capability of the proposed DPL model. [36,37]

The findings indicated that the DPL equation yields a more accurate estimation of results when compared to the Pence model. Furthermore, researchers estimated thermal damage to skin tissue using an analytical solution based on the DPL model. [8,38,39]. Research has shown that the thermal wave's speed increases as the temperature gradient's phase delay grows. Moreover, as the time delay (Ƭq) extends, local heat accumulation within the tissue also intensifies [8,[40], [41], [42]].

A comparative analysis of this model alongside the Fourier and heat wave models revealed that the dual-phase-lag heat transfer model, when coupled with blood flow effects, converges to the Fourier heat transfer equation—provided both time delays are either equal or zero [43,44].

In our research, we conducted a comparative study between the SLP of single-core nanoparticles and that of diamagnetic core-shell nanoparticles, being based on soft and hard magnetic ferrite phases. Additionally, we calculated the heat generated by mononuclear nanoparticles and core-shell nanoparticles, which find application in hyperthermia-based cancer treatment. Accurate prediction and control of tissue temperature distribution are crucial for effective cancer therapy. Furthermore, we explored various heat transfer models, including the DPL model. The DPL model allows us to capture microtissue responses at both temporal and spatial scales. Comparing the DPL method with other heat transfer approaches (such as the single-phase-lag (SPL) and Fourier models), we concluded that the DPL model aligns better with empirical results.

## 2 Theory and model

### 2.1 Magnetic hyperthermia

The mechanism underlying the relaxation of magnetic moments in nanoparticles emerges from the phenomenology of magnetic fluid hyperthermia (MFH). When magnetic nanoparticles disperse in a liquid, they exhibit rotational motion, which is closely tied to the concept of Brownian relaxation time.

(1) $$\tau_{B}=\frac{3\eta V_{H}}{k_{B}T}$$

η is environment viscosity, V_H_ is Hydrodynamic volume of nanoparticles, K _B_T is Thermal energy, K_B_ is Boltzmann constant.

Under the influence of heat-induced fluctuations, the magnetic torque gradually settles by surmounting an energy barrier associated with magnetic heterogeneity (referred to as Ea). This phenomenon becomes particularly evident during Néel's relaxation time [[45], [46], [47]].

(2) $$\tau_{N}=\tau_{0}e^{\frac{Ea}{k_{B}\cdot T}}$$

Ƭ_0_ is the spin relaxation time which is equal to 10^−9^s for a detailed analysis of Néel relaxation time [48].

Assuming that these two mechanisms are independent, the effective relaxation time is calculated by the relationship $\frac{1}{\tau }=\frac{1}{\tau_{N}}+\frac{1}{\tau_{B}}$ (3)

In this case, the dominant mechanism will be faster to produce heating.

Recent research suggests that optimizing heating efficiency involves adjusting the nanoparticle size to align with the total time corresponding to the applied frequency.

(4) $$2\pi \;v_{eff}\tau_{eff}=1$$

νeff is the Frequency For maximum heating [45,49,50].

Nanoparticles often absorb power, a phenomenon frequently explained using linear response theory (LRT).

### 2.2 Heat dissipation of MNPS

The power dissipation of MNPS is given by equation (5) [4]:

(5) $$Q_{np}=\pi \mu_{0}H_{0}^{2}f\ddot{\chi }$$

In the magnetic field of AC

(6) H=H0cos(⍵t)

Angular frequency ⍵ = 2πf (7) $\ddot{ꭓ}$ is the loss component of the magnetic susceptibility given by equation (8). ꭓ_0_ is the equilibrium susceptibility.

(8) $$\ddot{\chi }=\left[\frac{\omega \tau }{1+{\left(\omega \tau \right)}^{2}}\right]\chi_{0}=\left[\frac{2\pi f\tau }{1+{\left(2\pi f\tau \right)}^{2}}\right]\chi_{0}$$

Langevin equation [4].

(9) $$L\left(\xi \right)=\frac{M}{M_{s}}=\coth\left[\xi -\frac{1}{\xi }\right]$$

$\chi_{0}=\chi_{i}\frac{3}{\xi }\left[\coth\;\xi -\frac{1}{\xi }\right],\xi =\frac{\mu_{0}M_{d}HV_{M}}{k_{B}T}$ (10) ${ꭓ}_{i}$ is the initial susceptibility obtained

(11) $$\chi_{i}=\frac{\mu_{0}M_{d}^{2}\varphi V_{M}}{3k_{B}T}$$

τ is the effective relaxation time, τ_N_ is Neel relaxation, τ_B_ is Brownian relaxation time,

(12) $$\frac{1}{\tau }=\frac{1}{\tau_{N}}+\frac{1}{\tau_{B}}$$

η is the viscosity of nanofluid, T is the absolute temperature,

(13) $${\;\tau }_{N}=\frac{\sqrt{\pi }}{2}\tau_{0}\frac{e^{\Gamma }}{\sqrt{\Gamma }}$$

(1a) $$\tau_{B}=\frac{3\eta V_{H}}{k_{B}T}$$

(14) $$\Gamma =\frac{KV_{M}}{kT}$$

V_M_ is the volume of nanoparticles given by

(15) $${\;V}_{M}=\frac{\pi }{6}D^{3}$$

V_H_ is the hydrodynamic volume of MNP given by

(16) $$V_{H}=\frac{\pi }{6}{\left(D+2\delta \right)}^{3}$$

The Volume fraction of magnetic nanoparticles given by

(17) $$\varphi =\frac{M_{s}}{M_{d}}$$

**Fig. 1:**
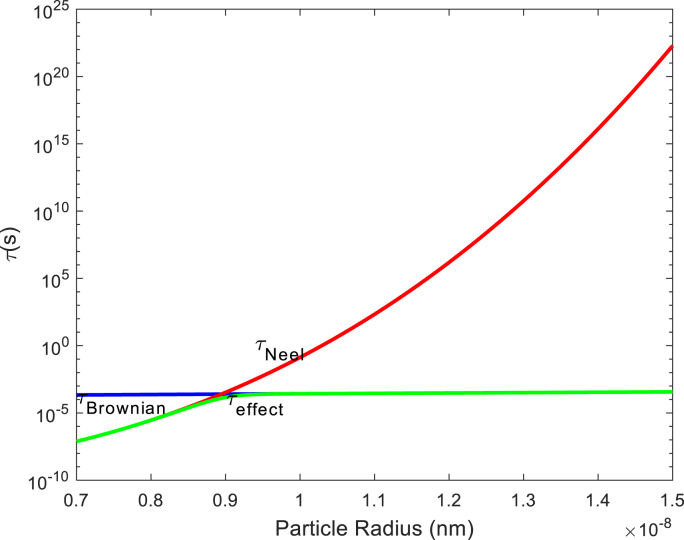
Ƭ(s) versus nanoparticle radius (nm).

Fig 1 is constructed using equations (1), (12), (13), (14), (15), (16). The power loss of nanoparticles in the presence of a constant heat source has been derived from equation (7). In this article, we introduce a novel method for harnessing heat from core-shell nanoparticles, leveraging the interplay between soft and hard magnetic ferrite phases.

## 3 Calculation of heat generated in terms of concentration and temperature

In adiabatic systems, the amount of heat released by nanoparticle power losses (SLP) [45].

According to Refs. [4,49]:

(18) $$SAR\;or\;SLP\left(\frac{W}{g}\right)=C\frac{\Delta T}{\Delta t}$$

(19) $$SLP=4.1868\pi \mu_{0}^{2}\frac{\varphi M_{s}^{2}V}{1000kT}\cdot H_{0}^{2}v\frac{2\pi v\tau }{1+{\left(2\pi v\tau \right)}^{2}}$$

(20) $$\text{SLP}=\frac{Qnp}{\rho np\;\varphi }$$

Substituting equation $\gamma_{np=\rho_{np}}$ φ [4] in above equation

We get

(21) $$Q_{np}=SLP\left(\rho_{np}\varphi \right)=SLP\left[\rho_{np}\;\left[\gamma_{np}/\rho_{np}\;\right]\right]$$

This indicates that the heat of nanoparticles is dependent on mass concentration.

(22) $$Q_{np}\left(\gamma_{np}\right)=\text{SLP}\left[\gamma_{np}\right]$$

Q_np_ in the above equation is volumetric power (w/m^3^)

(23) $$M_{S}\;\text{is}\;a\;\text{Magnetic}\;\text{saturation}\;\text{which}\;\text{is}\;\text{given}\;\text{by}\;\text{the}\;\text{formula}\;Ms=Ms_{b}{\left[\frac{\left(r-d\right)}{r}\right]}^{3}$$

M_sb_ is the Ms Volume, d is the surface thickness of the particles showing irregular rotations and r is the radius of nanoparticles

(24) $$V=\frac{4}{3}\;\pi r^{3}$$

V is the magnetic volume for a particle with a radius of r, H0 is Magnetic field strength, ν is Oscillating magnetic field frequency and τ_eff_ is relaxation time.

(25) $$\tau_{N}=\tau_{0}e^{\frac{k\cdot V}{k_{B}\cdot T}}$$

$\tau_{B}=\frac{3\eta V_{B}}{K_{B}\cdot T}$ (1) T is Sample temperature, Ƭ_0_ is non-reciprocal relaxation time of magnetic nanoparticles (10^−9^ s-10^−12^ s), K is Anisotropy constant (23000–100000 j/m^3^), η is Viscosity of the surrounding liquid, V_H_ is Hydrodynamic volume of the particle, ρ_mnp_ is density of nanoparticles (see Table 1) [4,49,51].

Referring to Table 2, when a soft ferromagnetic material is employed in the core and a hard ferromagnetic material in the shell, the specific loss power (SLP) value tends to be higher. The power dissipation by magnetic nanoparticles, whether from a constant or variable heat source, can be determined using equation (22). Similarly, the power loss of magnetic nanoparticles listed in the table above can also be obtained from the same equation (see Table 3).

**Table 1:** Parameters used in the phenomenon of heating [4,51].

Parameter	Description	Values	Units	Source
D	Diameter of Nps	15	Ηm	]4,51[
δ	Legend layer	1	Ηm	]4,51[
H_0_	Magnetic field Intensity	37.4	kA/m	]51[
f _0_	The frequency of magnetic field	500	KHz	]4,51[
M_d_	Domain magnetization	6.3 × 610^5^	A/m	]51[
μ_0_	The permeability of free space	4π × 10^−7^	N/A^2^	]4,51[
$K_{B}$	Boltzmann constant	1.38 × 10^−23^	J/k	]4[
k	Thermal conductivity	5.3 × 10^6^	w/m/k	]4,51[
K	Magnetic anisotropy constant	1.5 × 10^4^	J/m^3^	]4,51[
Η	Dynamic viscosity of nanofluid	1.22 × 10^−3^	Pa.s	]4 [
φ	Volume fraction of Nps	0.0013	–	]4,51[

**Table 2:** Properties of different MNPs with equal radius(r = 15 nm) [14,49].

Core	Shell	Amplitude, H_0_(kA/m)	frequency(kHz)	f.H_0 (_A/ms)	SLP(w/gr)
MnFe2o4	–	37.3	500	10^9^ × 18	411
MnFe2o4	CoFe2o4	37.3	500	× 1810^9^	3034
CoFe2o4	MnFe2o4	37.3	500	10^9^ × 18	2274.12
Zn_0.4_Co_0.6Fe2o4_	Zn.4Mn.6Fe2o4	37.3	500	10^9^ × 18	3866

**Table 3:** Parameters used in bioheat and SPL and DPL model and properties of the liver [4].

Parameter	Description	Values	Units
T_b_	Arterial blood temperature	37	ᶱ C
C _b_	Specific heat of blood	3500	j/kg/k
$\omega_{b}$	Blood perfusion rate	0.0151	1/s
ρ _b_	Density of blood	1050	Kg/m^3^
Q _met_	Metabolic heat	10713	w/m^3^
C _ν_	Heat capacity	3500	j/kg/k
K	Thermal conductivity	0.642	w/m/k

## 4 Heat transfer in liver tissue

One method of heat transfer involves utilizing Pence's biological heat transfer equation, which is based on Fourier's law. According to this model, heat diffusion occurs instantaneously and is directly related to the heat flux (q).

(26) $$q\left(\vec{r,}t\right)=-k\nabla T\left(\vec{r,}t\right)\text{.}$$

Pence's biological heat transfer equation, based on energy conservation in the Cartesian coordinate system, can be expressed as follows:

(27) $$\rho c\frac{\partial T}{\partial t}={k\nabla }^{2}T-\omega_{b}\rho_{b}c_{b}\left(T-T_{b}\right)+Q_{m}+Q_{np}$$

The thermal delay, associated with the time delay between heat flux and temperature gradient, is introduced in Fourier's conduction law: [6, 26, 52]

(28) $$q\left(\vec{r,}t+\tau_{q}\right)=-k\nabla T\left(\vec{r,}t\right)\text{,}$$

In this context, Ƭ q represents the thermal relaxation time—a measure of the time delay between the heat flux vector and the temperature gradient. By combining the time phase delay associated with thermal relaxation (which extends Fourier's law linearly and also considers non-Fourier heat transfer models) with the single-phase delay (SPL), we arrive at what is known as the Cattaneo-Vernotte (C–V) heat transfer equation. The SPL thermal equation takes a hyperbolic form and can be expressed using the following equation:

(29) $$\tau_{q}\rho c\frac{\partial^{2}t}{\partial t^{2}}+\left(\rho c+\tau_{q}\omega_{b}\rho_{b}c_{b}\right)\frac{\partial T}{\partial t}=k\nabla^{2}T-\omega_{b}\rho_{b}c_{b}\left(T-T_{b}\right)+Q_{m}+Q_{np}+\tau_{q}\left(\frac{\partial Q_{m}}{\partial t}+\frac{\partial Q_{np}}{\partial t}\right))$$

Tzou [6,53] proposed the consideration of an additional relaxation time to explore the effects of microstructural interactions and rapid thermal phenomena. Consequently, the relationship between heat flux and temperature gradient underwent modification as follows.

(30) $$q\left(\vec{r,}t+\tau_{q}\right)=-k\nabla T\left(\vec{r,}t+\tau_{t}\right)$$

Ƭq is the Phase delay for heat flux, Ƭt is the Phase delay for temperature gradient.

Consequently, when Ƭq < Ƭt, the heat flux leads the temperature gradient; conversely, when Ƭq ≥ Ƭt, the temperature gradient precedes the heat flux [6,24]. By replacing equation (27) in equation (30) and performing necessary mathematical operations, we arrive at the non-Fourier heat transfer model with dual-phase-lag (DPL). The temperature field within the tissue can be calculated using the following equation, which represents the non-Fourier heat transfer model with dual-phase-lag:

$$\tau_{q}\rho c\frac{\partial^{2}T}{\partial t^{2}}+\left(\rho c+\tau_{q}\omega_{b}\rho_{b}c_{b}-\tau_{t}k\nabla^{2}\right)\frac{\partial T}{\partial t}=$$

(31) $$k\nabla^{2}T-\omega_{b}\rho_{b}c_{b}\left(T-T_{b}\right)+Q_{m+}Q_{np}+\tau_{q}\left(\frac{\partial Q_{m}}{\partial t}+\frac{\partial Qnp}{\partial t}\right)\text{.}$$

For Ƭ_t_ = 0, Equation (31) simplifies to Equation (29) from the SPL biological heat transfer model. Similarly, when Ƭq = 0, Equation (31) further reduces to Equation (27), which corresponds to the Fourier heat transfer equation.

C is the Specific heat capacity (J/kg·°K),T is the Tissue temperature, Kis the Heat conductivity, ρb is the Blood density, Cb is the Specific heat capacity of blood (J/kg·°K),ωb is Blood perfusion ratio (1/s),Tb is the Temperature of the blood entering the tissue.

The expression [ρb Cb ωb (T - Tb)] captures the impact of heat reduction due to small blood vessels.

Q _m_: Represents metabolic heat (W/m^3^).

Q_np_: Describes volumetric heat generation in thermal ablation processes, such as radio frequency, laser heat sources, and magnetic nanoparticles. Specifically, it analyzes the heat produced by magnetic nanoparticles under the influence of a magnetic field.

**Fig. 2:**
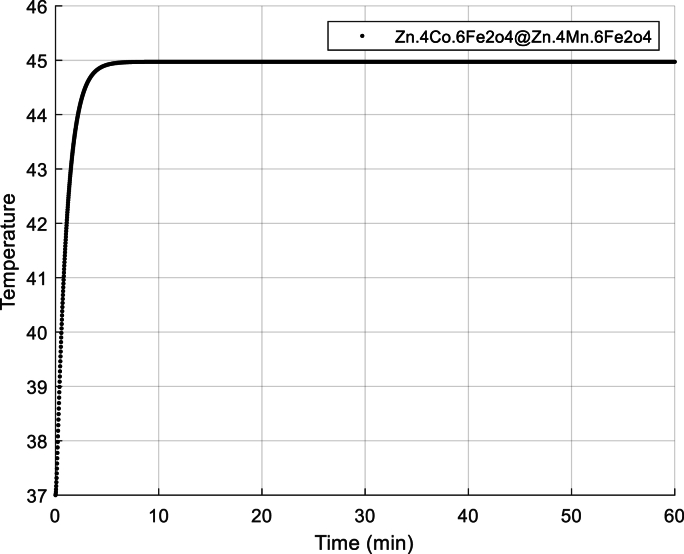
Temperature versus time diagram.

**Fig. 3:**
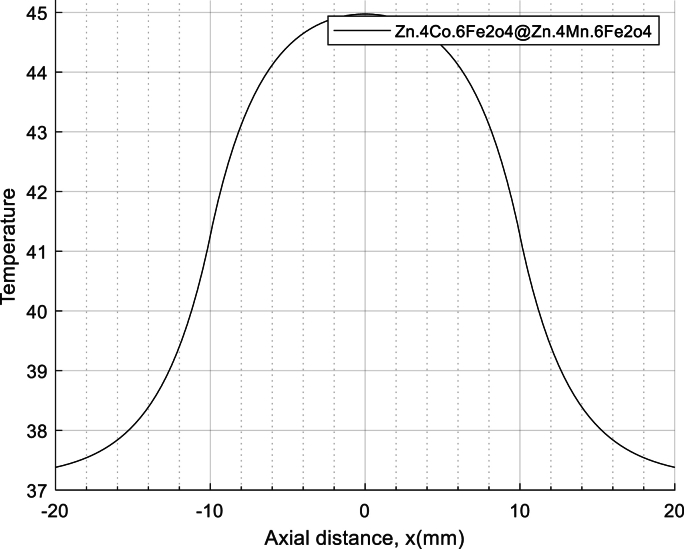
Fig. 3.Temperature distribution based on the X-coordinate for the nanoparticle Zn_0.4_Co_0.6_Fe_2_O_4_@Zn_0.4_Mn_0.6_Fe_2_O_4_ in model DPL.

**Fig. 4:**
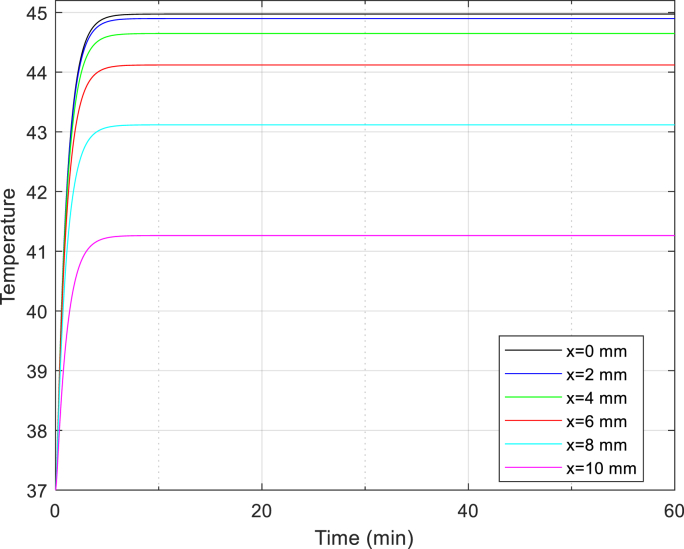
Fig. 4 Temperature distribution over time in liver tissue after 60 min in DPL model.

The concept of normal thermal texture is defined by Equation (31), specifically the dual-phase non-Fourier heat transfer equation (DPL). Lee's experiments on liver tissue provide valuable insights: Ƭq = 5.66 s and Ƭt = 22 s are the relevant time constants. Notably, the phase delay of heat flux in the SPL model corresponds to Ƭq = 5.66 s (as documented in reference]54[). Equation (31) also allows us to calculate the temperature range within biological tissue. Additionally, visual representations aid our understanding: Fig **2** illustrates the temperature variation over time, while Fig **3** depicts the temperature distribution along the axial distance for magnetic nanoparticles. (Zn_0.4_Co_0.6_Fe_2_o_4_@Zn_0.4_Mn_0.6_Fe_2_o_4_ with the DPL method, calculated by equation (31)). Fig **4** depicts the temperature distribution over time in liver tissue after 60 min, as calculated using the DPL model based on Equation (31).

**Fig. 5:**
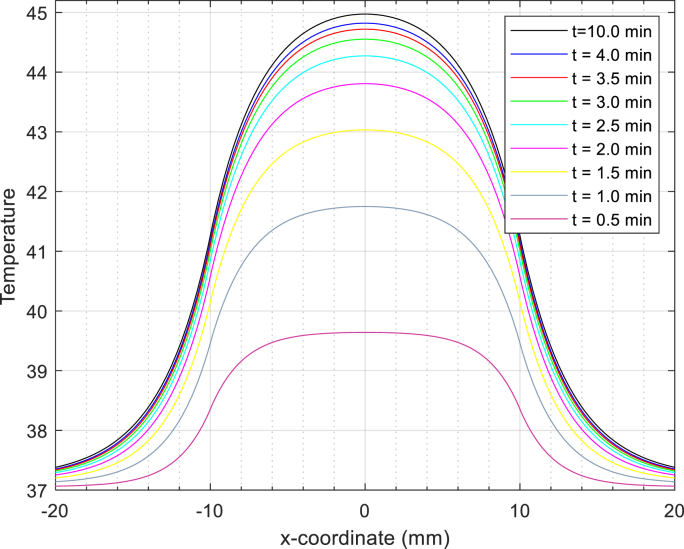
Fig. 5. Temperature distribution in liver tissue. The temperature curve is based on X coordinates for various selected times.

In Fig **5**, we observe the temperature distribution along the X-coordinates at various selected time points within liver tissue. This distribution is modeled using the DPL approach, with calculations based on Equation (31). Moving on, Fig **7 and 8** simulate heat transfer in the DPL model, considering different magnetic nanoparticles within a magnetic field. Again, Equation (31) serves as the foundation for these simulations. For a broader perspective, Fig **9 and 10** provide a comparative analysis of heat transfer methods: Fourier, SPL, and DPL. These comparisons are based on Equations (27), (29), (31), respectively.

As for the initial conditions, we assume that the tumor's temperature equals that of normal tissue (T = T_0_ = 37 °C). Additionally, the boundary conditions incorporate thermal insulation.

(32) −n·(−k∇T)=0

Adjacent tissue is with one needle. The liver's external boundary conditions are as follows.

(33) $$-n\cdot \left(-k\nabla T\right)=h\left(T_{ext}-T\right)$$

h = 200 w/K/m^2^ is the heat transfer coefficient] 4[

## 5 Results and discussions

In this article, we employ the finite volume method (FVM) to solve a captivating puzzle: simulating heat distribution in liver tissue and achieving tumor destruction.

To tackle this challenge, we explore several heat transfer models, including the classical Fourier equations, singular phase Lag (SPL), dual-phase lag (DPL), and the behavior of magnetic core-shell nanoparticles under the influence of an AC magnetic field. In our study, we highlight the most effective core-shell nanoparticles—those that exhibit superior heat production compared to their soft and hard counterparts. We then incorporate the heat generated by these exceptional nanoparticles directly into our heat transfer equations. Through rigorous comparisons, we unveil the optimal method: the dual-phase delay approach. Not only does it align better with experimental data, but it also proves adaptable for use in heterogeneous tissues. For our simulations, we rely on numerical methods due to their heightened accuracy and the elegant simplicity of their mathematical formulations.

The numerical parameters used are as follows:

The DPL model incorporates two distinct phase delays: one for the heat flux (Ƭ q) and another for the temperature gradient (Ƭt). Lee's experiments on liver tissue provide valuable insights: Ƭq = 5.66 s and Ƭt = 22 s are the relevant time constants. Notably, the phase delay of the heat flux in the SPL model is also Ƭq = 5.66 s [8,54].

Within our model, the tumor is treated as a solid body. We deliberately disregard the tumor's elasticity and any deformation caused by fluid flow pressure. Here's how it unfolds:

1. **Nanoparticle Injection**: We introduce diluted nanoparticles into the tissue. When magnetic nanoparticles (MNPs) are injected under externally applied pressure, an intriguing phenomenon occurs. Within approximately 15 min (depending on the flow rate), the entire nanofluid infiltrates the tissue. Picture it: the nanofluid creating a needle-like channel under liquid pressure, initiating the release of nanoparticles within the tumor.
2. **Distribution and Penetration**: Over the next 24 h, the nanofluids disperse throughout the tumor region. Interestingly, while a small portion of the nanofluid manages to penetrate healthy tissue, the majority of nanoparticles find their way into the tumor area post-release. This gradual process—typically taking 24 h—sets the stage for the next step.
3. **Heat Transfer Investigation**: Now, our focus shifts to heat transfer within liver tissue. We achieve this by applying an alternating current (AC) magnetic field to the nanoparticles. As these particles respond to relaxation effects (think Néel relaxation and Brownian motion), they begin to vibrate and generate heat.

Fig. 1: **Ƭ(s) is drawn according to the radius of the nanoparticles.**Fig. 1 shows the effects of $\tau_{Neel}$ , $\tau_{Brownian}$ and $\tau_{effect}$ for the nanoparticles with a radius ranging from 7 to 15 nm. Magnetic nanoparticles (MNPs) vibrate and generate heat due to Néel and Brownian relaxation. This heat, known as “Lost Heat” (SLP), raises the temperature within the tumor. Importantly, this heat source plays a role in various models, including DPL, SPL, and bioheat models.

Before the magnetic field is applied, the temperature in both the tumor and normal tissue is 37 °C. However, once the magnetic field takes effect, the tissue temperature begins to rise. Within the initial 10–15 min, it climbs to 45 °C, a remarkable warming period. Notably, at the very center of the tumor, the temperature peaks and then gradually decreases radially toward the outer edges.

Fig. 2. The temperature-time graph reveals a fascinating trend: as time progresses, the temperature steadily rises. This phenomenon occurs because the external magnetic field prompts the nanoparticles to vibrate and release heat. The amount of heat for this nanoparticle has been obtained as Q _np=_10^5^ × 4.77978w/m^3^

Fig. 3. The simulation of temperature changes along the X-axis for specific time intervals reveals an interesting trend: as time progresses, the temperature consistently increases. Remarkably, this pattern aligns excellently with the simulation results for X-coordinates ranging from 0 to 20.

To validate the temperature curve, we have plotted data based on X-coordinates ranging from −20 to 20 (mm).

Fig. 3 conforms to a Gaussian distribution curve and has undergone validation through simulation by K. Adhikary et al. [4,55]. This alignment suggests that the temperature-X plot corresponds excellently with the experimental data.

Fig. 4 Temperature distribution over time in liver tissue after 60 min in DPL model.

This Fig. depicts the two-dimensional temperature distribution within liver tissue, specifically assessing tumor temperatures at selected points along the X-axis (X j: j = 0, 2, 4, 6, 8, and 10 mm). This visualization provides valuable insights. Additionally, the temperature-time graph has been simulated for these specific points.

After 3600 s, the temperature at x = 0 is 45 °C, while at x = 10 mm, it reaches 40.8 °C. This observation highlights that as we move away from the tumor center, the temperature gradually decreases. The highest temperature occurs precisely at the tumor center, whereas the lowest temperature is found at the boundaries of the liver tissue.

Fig. 5. shows changes in the temperature in the coordinates X at selected times t = 0.5,...,10 min are shown in Fig. 5.

**Fig. 6:**
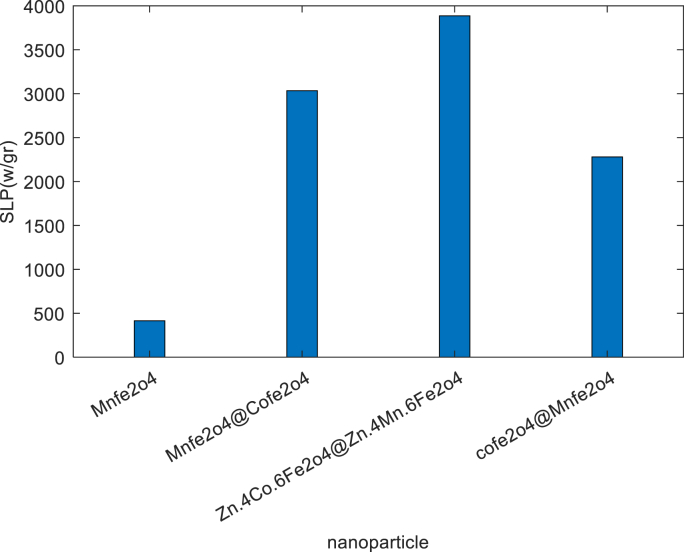
Bar chart SLP (w/gr) based on the nanoparticles.

The temperature-x-coordinate curve exhibits a Gaussian distribution, symmetric around the y-axis. Notably, the temperature reaches its maximum value precisely at the center of the tumor, gradually decreasing radially toward the outer regions. As time progresses, the temperature curves ascend to higher values—from t = 0 min up to 9 min. Remarkably, after 60 min, the peak temperature remains centered within the tumor. These curves continue their radial descent, reaching their minimum value at the outer border of the liver. This shows the superiority of the Zn_0.4_ Co_0.6_ Fe_2_ O_4_ @Zn_0.4_ Mn_0.6_ Fe_2_ O_4_ nanomagnetic core-shell configuration, with an impressive specific loss power (SLP) of 3866w/g [49] This nanomagnetic design outperforms several other core-shell and homogeneous nanomagnetic structures.

Several nanoparticles with identical radii have been compared in terms of their specific loss power (SLP) (see Fig. 6).

The graph reveals an intriguing trend: core-shell magnetic nanoparticles outperform their homogeneous counterparts in terms of specific loss power (SLP). Additionally, the soft-hard nanoparticle configuration exhibits higher SLP values compared to hard-soft arrangements. Specifically, when considering nanoparticles with the same radius [49].

• MnFe_2_O_4_@CoFe_2_O_4_: Achieves an SLP of 3034 W/g.
• MnFe_2_O_4_: Yields an SLP of 411 W/g.
• CoFe_2_O_4_@MnFe_2_O_4_: Attains an impressive SLP of 2274.12W/g.
• This shows the superiority of the Zn_0_._4_Co_0_._6_Fe_2_O_4_@Zn_0_._4_Mn_0_._6_Fe_2_O_4_ nanomagnetic core-shell configuration, with an impressive specific loss power (SLP) of 3866 W/g. This nanomagnetic design outperforms several other core-shell and homogeneous nanomagnetic structures.

The exchange pair plays a crucial role in influencing the surface phenomenon. By increasing the number of interface exchange pairs, we can enhance the specific loss power (SLP). Specifically, the exchange pair parameter represents the average value between two distinct areas [14,49].

The thermomagnetic response undergoes modulation through the doping of nanoparticles’ cores with Zn^2+^ diamagnetic ions. Interestingly, the introduction of zinc weakens super-exchange interactions, resulting in an increased thermomagnetic coefficient (representing magnetism per unit of mass) and a reduction in the Curie temperature. This deliberate adjustment ensures that the nanoparticles achieve a safe temperature range of 41–45 °C [45,[57], [58], [59], [60], [61], [62]]. Furthermore, the addition of Zn not only decreases interaction rates but also induces energy absorption [14]. Beyond these factors, the combined effects of cobalt, zinc, and iron atoms prove remarkably effective. Among the various nanoparticles, we spotlight Zn_0_._4_Co_0_._6_Fe_2_O_4_@Zn_0_._4_Mn_0_._6_Fe_2_O_4_, boasting an impressive specific loss power (SLP) of 3866 W/g, as the optimal choice for cancer treatment.]14,49[

**Fig. 7:**
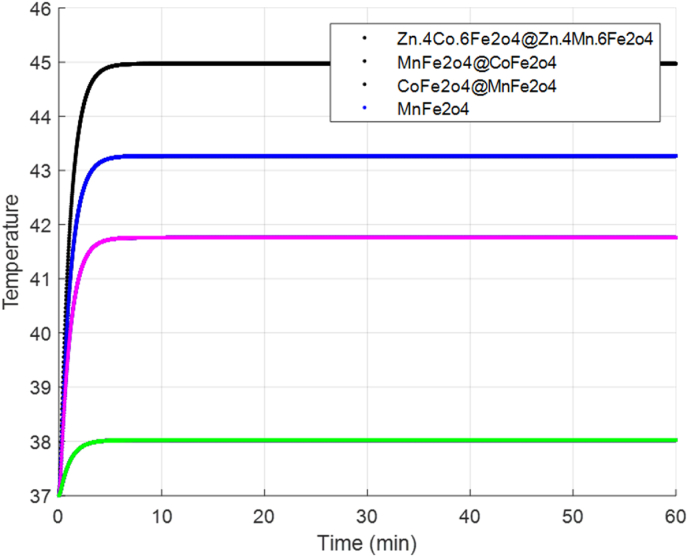
The temperature versus time graph over a 60- minute period in the DPL model

Fig. 7. Temperature distribution curves resulting from various heat effects using different nanoparticles in a magnetic field are depicted over a 60- minute period in the DPL model. Notably, the highest temperature is associated with Zn_0_._4_Co_0_._6_Fe_2_O_4_@Zn_0_._4_-Mn_0_._6_Fe_2_O_4_, while the lowest temperature corresponds to MnFe_2_O_4_.

**Fig. 8:**
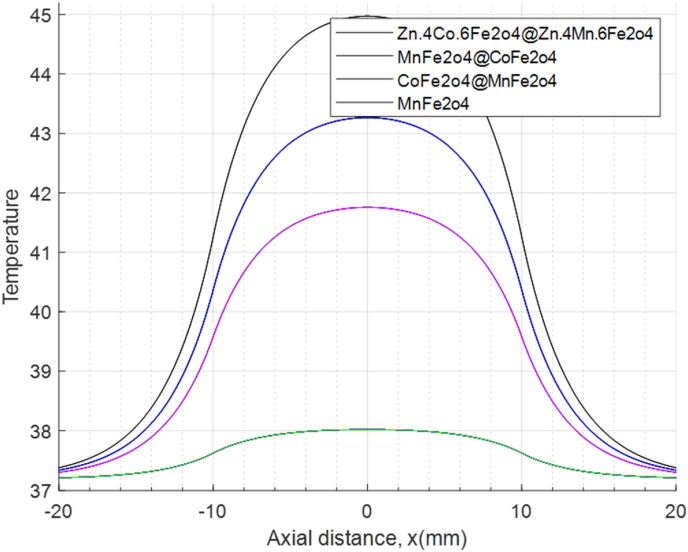
The temperature versus X-coordinate graph in the DPL model

Fig. 8. In the context of the DPL model, temperature distribution curves resulting from various heat effects with different nanoparticles in a magnetic field are plotted against X-coordinate curves. Notably, the highest temperature is associated with Zn_0_._4_Co_0_._6_Fe_2_O_4_@Zn_0_._4_–Mn_0_._6_Fe_2_O_4_, while the lowest temperature corresponds to MnFe_2_O_4_.

This article explores three heat transfer methods—Fourier, SPL, and DPL—specifically applied to the Zn_0_._4_Co_0_._6_Fe_2_O_4_@Zn_0_._4_Mn_0_._6_Fe_2_O_4_ core-shell nanoparticle.

**Fig. 9:**
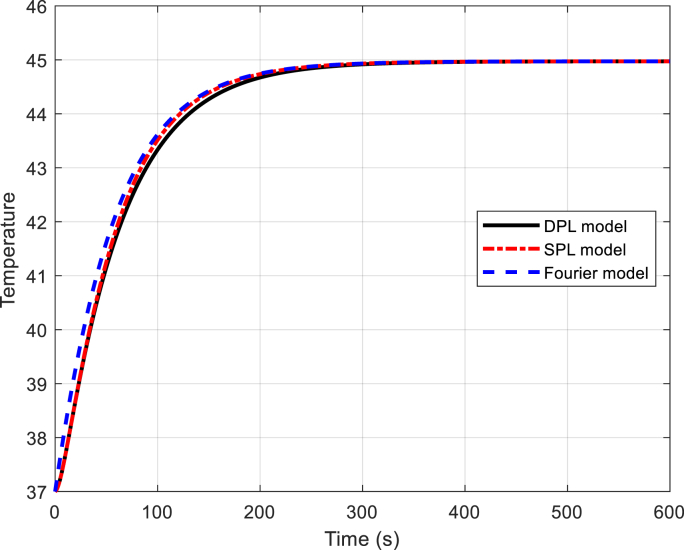
The temperature versus time for DPL, SPL and Fourier model.

Fig. 9. The temperature distribution curve over time for the nanoparticle Zn_0_._4_Co_0_._6_Fe_2_O_4_@Zn_0_._4_Mn_0_._6_Fe_2_O_4_ is depicted across three models: Fourier, SPL, and DPL, at a distance of 1.5 mm. Fig. 9 reveals that the DPL model exhibits a maximum temperature lower than both the Fourier and SPL models. This discrepancy can be attributed to the limited speed of heat spread within the DPL model compared to other methods.

**Fig. 10:**
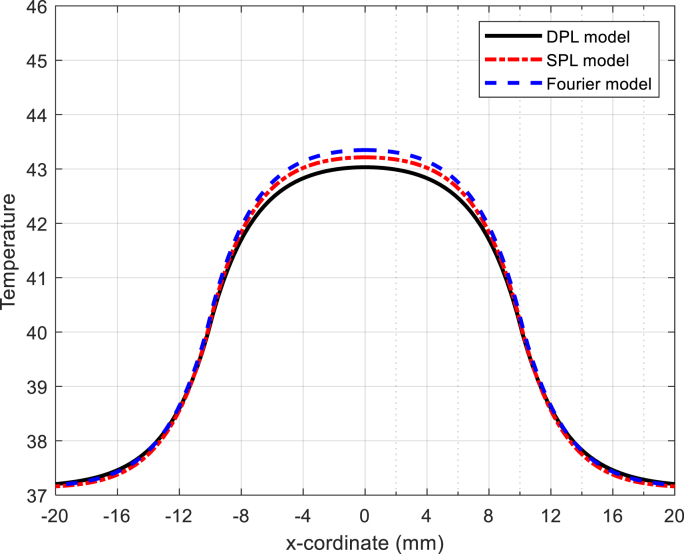
the temperature versus X-coordinate for DPL , SPL and Fourier model.

Fig. 10. The temperature distribution curve along the x coordinates for the nanoparticle Zn_0_._4_Co_0_._6_Fe_2_O_4_@Zn_0_._4_Mn_0_._6_Fe_2_O_4_ is displayed across three models (Fourier, SPL, and DPL) after 300 s. Notably, the DPL model provides a more accurate estimation of real temperature compared to both the SPL and Fourier models in t_f_ = 600s.

**Fig. 11:**
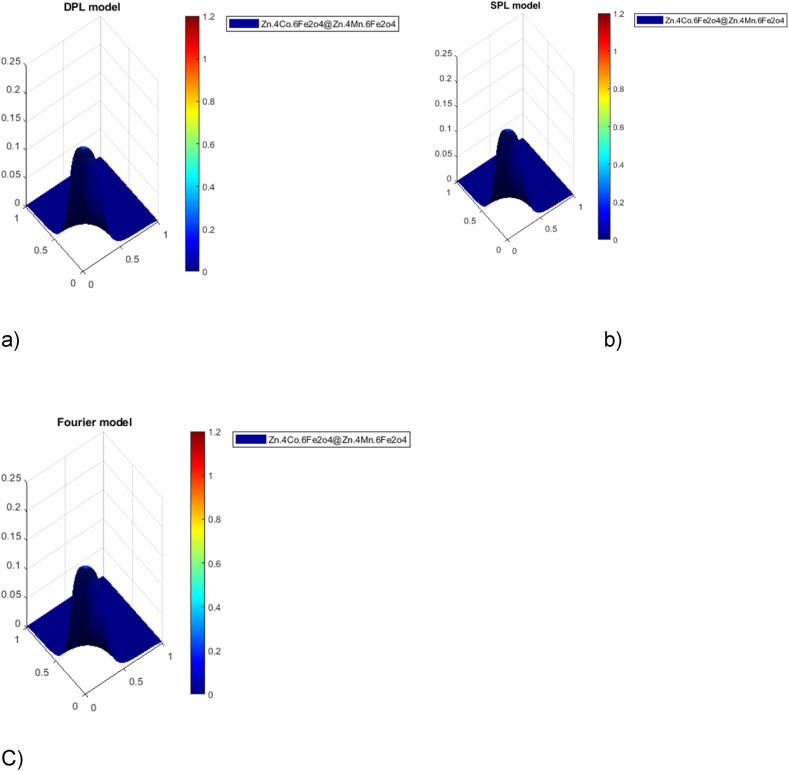
Surface temperature distribution non-dimensional plots in tumor tissue and healthy liver considering non-dimensional spatial coordinates in the a) DPL model, b) SPL model and c) Fourier model for nanoparticle Zn_0.4_Co_0.6_Fe_2_O _4 @_Zn_0.4_Mn_0.6_Fe_2_O_4_.

In the non-Fourier DPL model, the temperature profile gradually decreases as anticipated, owing to the delayed behavior of thermal radiation—a phenomenon that takes time. Remarkably, the DPL model's estimations align more closely with empirical results than alternative methods. When we apply the DPL model, the maximum temperature decreases, leading to a reduction in the necrotic area within the tumor. At the microtissue scale, the DPL model adeptly captures responses over both time and space. Furthermore, when compared to other methods, estimating thermal damage using the DPL model yields more accurate results, consistent with experimental observations. The assumption of infinite heat propagation speed, often invoked in simpler models, doesn't hold in the complex tissue structure. To account for the real behavior of tissues, we must consider non-Fourier heat conduction models. Phase delay values obtained from experiments (such as those conducted by Lee et al. on liver tissue) provide valuable insights. Notably, the DPL model emerges as a superior estimator of thermal damage. This estimation bears critical importance in clinical practice, where achieving maximal tumor tissue destruction while sparing healthy tissue remains the ultimate goal.Fig. 11

## 6 Conclusion

In this article, we investigate the impact of time delays associated with the phases of the Fourier, single-phase lag (SPL), and dual-phase lag (DPL) models. Our focus is on simulating heat distribution in biological liver tissue using core-shell magnetic nanoparticles under an alternating current (AC) magnetic field. These nanoparticles hold promise for local hyperthermia treatment of liver cancer. To select the most effective magnetic nanoparticle, we employ core-shell structures based on both soft and hard magnetic ferrites, considering the combined effects of cobalt, zinc, and iron. Specifically, we compare the SLP of single-core nanoparticles composed of MnFe_2_O_4_ with that of core-shell nanoparticles, including MnFe_2_O_4_@CoFe_2_O_4_, CoFe_2_O_4_@MnFe_2_O_4_, and Zn_0_._4_Co_0_._6_Fe_2_O_4_@Zn_0_._4_Mn_0_._6_Fe_2_O_4_. Our findings reveal that the SLP of core-shell double-core magnetic nanoparticles surpasses that of single-core nanoparticles. Furthermore, among nanoparticles with two magnetic cores, those with a soft ferromagnetic core and a hard ferromagnetic shell exhibit higher SLP compared to their counterparts with a hard ferromagnetic core and a soft ferromagnetic shell. Specifically, we obtained SLP values of 3034 W/g and 2274.12 W/g for MnFe_2_O_4_@CoFe_2_O_4_ and CoFe_2_O_4_@MnFe_2_O_4_, respectively, as double-core magnetic nanoparticles. Notably, among the materials considered, Zn_0_._4_Co_0_._6_Fe_2_O_4_@Zn_0_._4_Mn_0_._6_Fe_2_O_4_ demonstrates the highest SLP value, reaching 3866 W/g.

We have also determined that specific combinations enhance the properties of MNPs, resulting in higher SLP values and increased power dissipation compared to individual magnetic nanoparticles such as MnFe_2_O_4_, MnFe_2_O_4_@CoFe_2_O_4_, and CoFe_2_O_4_@MnFe_2_O_4_. Consequently, Zn_0_._4_Co_0_._6_Fe_2_O_4_@Zn_0_._4_Mn_0_._6_Fe_2_O_4_—when used for tumor destruction—proves more effective and leads to improved treatment outcomes. This enhanced efficacy likely arises from a hardening effect. Further investigation reveals that the strengthening of the soft magnetite shell results from the combined influence of cobalt, zinc, and iron atoms, particularly in nanoparticles with larger core sizes. Moreover, our research demonstrates that the unique core-shell structure significantly enhances heat generation in response to an AC magnetic field.

In our study, we simulated the temperature distribution resulting from the diverse heat effects of various core-shell nanoparticles within an alternating current (AC) magnetic field, specifically along the T-t coordinates. Additionally, we examined the time-dependent temperature distribution caused by these heat effects over a 60-s interval using the dual-phase lag (DPL) model. Among the investigated nanoparticles, the highest temperature was associated with Zn_0_._4_Co_0_._6_Fe_2_O_4_@Zn_0_._4_Mn_0_._6_Fe_2_O_4_, while the lowest temperature corresponded to MnFe_2_O_4_. Our findings suggest that these specific combinations maximize the properties of magnetic nanoparticles (MNPs), resulting in higher specific loss power (SLP) values and greater overall power dissipation compared to other magnetic nanoparticles, such as MnFe_2_O_4_, MnFe_2_O_4_@CoFe_2_O_4_, CoFe_2_O_4_@MnFe_2_O_4_, and Zn_0_._4_Co_0_._6_Fe_2_O_4_@Zn_0_._4_Mn_0_._6_Fe_2_O_4_. This enhanced performance in hyperthermia cancer treatment holds promise for more effective clinical outcomes.

The simulation of temperature gradients over time for Zn_0_._4_Co_0_._6_Fe_2_O_4_@Zn_0_._4_Mn_0_._6_Fe_2_O_4_ using the dual-phase lag (DPL) model reveals an interesting trend. As time progresses, the temperature steadily increases. This phenomenon occurs because the external magnetic field induces vibrations in the magnetic nanoparticles precisely at the tumor site, leading to the release of heat.

Additionally, we explore the temperature distribution based on nanoparticle location. Specifically, we focus on Zn_0_._4_Co_0_._6_Fe_2_O_4_@Zn_0_._4_Mn_0_._6_Fe_2_O_4_ within the DPL model during selected time intervals. Notably, as time elapses, the temperature values rise. For radial distances ranging from 0 to 20, our simulations align remarkably well with the temperature profiles along the X coordinates within the same range. To validate our findings, we've plotted temperature curves for coordinates spanning from −20 to 20.

The temperature distribution plot exhibits a Gaussian curve, a validation supported by simulations conducted by K. Adhikary et al. [4,55]. This alignment between the simmulated plot and experimental data underscores its accuracy. In our study, we simulated the two-dimensional temperature distribution over time within liver tissue using the dual-phase lag (DPL) model. Our focus was on quantitatively evaluating tumor temperature at specific points along the X-axis (X_j:j = 0,2,4,6,8,10 mm). After 60 min, we observed that the temperature at x = 0 reached 45 °C, while at x = 10 mm, it stabilized at 40.8 °C. Notably, as we move away from the tumor center, the temperature gradually decreases. The highest temperature occurs precisely at the tumor center, while the lowest is found at the boundaries of the liver tissue. Furthermore, in another analysis (depicted in “Fig 5”), we explored temperature distributions based on the X-coordinate at different time points within liver tissue using the non-Fourier DPL heat transfer model.

When we simulate the temperature distribution along the x-coordinates within liver tissue using the DPL model at various time points, an interesting pattern emerges. The temperature curve closely resembles a Gaussian distribution, symmetric around the y-axis. After 60 min, we observe that the maximum temperature occurs precisely at the tumor center. As we move radially outward, the temperature gradually decreases, ultimately reaching a minimum at the outer border of the liver.

The simulation presented here draws upon empirical data and results reported in references [4, 8, [54], [55], [56]] Our study aims to explore various heat transfer models, including both Fourier and non-Fourier approaches, while considering the impact of temporal and spatial phase delays inherent in the DPL model. We specifically investigate these models within both homogeneous and heterogeneous tissues. An external alternating magnetic field significantly influences magnetic core-shell nanoparticles.

These nanoparticles contribute to heat generation, which we incorporate into the heat transfer equation. Our analysis involves estimating temperature distributions using three distinct methods: Fourier, single-phase lag (SPL), and DPL. Remarkably, our findings lead us to conclude that the DPL model is the most effective approach. Notably, it allows for tumor destruction while preserving the health of surrounding tissue.

## Fundings

No financial support or funding was provided for the conduct of this research or preparation of the article.

## Declaration of competing interest

The authors declare that they have no known competing financial interests or personal relationships that could have appeared to influence the work reported in this paper.
